# Determinants of late antenatal care presentation in rural and peri-urban communities in South Africa: A cross-sectional study

**DOI:** 10.1371/journal.pone.0191903

**Published:** 2018-03-08

**Authors:** Joy Ebonwu, Alexandra Mumbauer, Margot Uys, Milton L. Wainberg, Andrew Medina-Marino

**Affiliations:** 1 Research Unit, Foundation for Professional Development, Pretoria, South Africa; 2 Integrated Health Systems Strengthening Department, Foundation for Professional Development, Pretoria, South Africa; 3 New York State Psychiatric Institute, Columbia University, New York, United States of America; University of Washington, UNITED STATES

## Abstract

**Objective:**

To investigate and compare determinates for delayed first presentation to antenatal care (ANC) services.

**Methods:**

A cross-sectional study was conducted amongst pregnant women attending their first ANC visit in rural Capricorn District and peri-urban Tlokwe sub-district communities in South Africa. Data collection included questionnaires and medical record abstraction. Bivariate and multivariate analyses assessed factors associated with late ANC presentation.

**Results:**

We recruited 807 pregnant women. Of these, 51% of rural women and 28% of peri-urban women presented late for first ANC. Rural women were more likely to present late for first ANC (AOR = 2.65; 95% CI 1.98–3.55) and report barriers to accessing ANC services (*P*<0.0001). Late ANC presentation in rural communities was associated with being married (AOR = 2.36; 95% CI 1.33–4.19), employed (AOR = 1.90; 95% CI 1.03–3.50), <20 years of age (AOR = 2.19; 95% CI 1.10–4.37), and reporting an unplanned pregnancy (AOR = 2.21; 95% CI 1.40–3.50). Late presentation in peri-urban communities was associated with unplanned pregnancy (AOR = 1.67; 95% CI 1.01–2.74), being told to come back later to initiate ANC after presenting early (AOR 0.51; 95% CI 0.30–0.89) and being pregnant for the first time (AOR = 0.56; 95% CI 0.34–0.94)

**Conclusion:**

Both rural and peri-urban women had high rates of late presentation for first ANC. However, women in the rural communities were more likely to present late. Unplanned pregnancy was an independent risk factor in both rural and peri-urban communities. Interventions around family planning, especially for adolescent girls and young women, are needed to improve early presentation for ANC.

## Introduction

Antenatal care (ANC) includes preventive and curative care services delivered during pregnancy. During ANC, health providers monitor for and identify risk factors relating to poor maternal and birth outcomes. Once identified, providers can initiate appropriate medical and educational interventions to reduce risks for maternal-neonatal morbidity and mortality. ANC services, especially at first visit, includes essential screening for health conditions such as human immunodeficiency virus (HIV) and Syphilis; for HIV-infected pregnant women, the maximum benefit of antiretroviral therapy (ART) to prevent mother-to-child transmission (PMTCT) of HIV requires early presentation to the health system [1]. Furthermore, immunizations, such as Tetanus toxoid, given during pregnancy can be life-saving for both mother and infant.

Though the World Health Organization (WHO) recommends initiation of ANC during the first trimester, late first ANC presentation remains widespread in sub-Saharan Africa, with geographical differences in the timing of ANC presentation between urban and rural settings identified within countries [2,3]. Documented risk factors for late first ANC presentation include lack of education, attitudes and knowledge regarding pregnancy, being unmarried, history of obstetric complications, and cultural beliefs. Availability, affordability and accessibility of health care services have also been identified as risk factors for late ANC presentation [4–9]. Booking delays, where women present early to the clinics but are given appointment to initiate ANC at a later date, have been reported in South Africa [10]. The ideal, however, is for ANC to be initiated when women first present to the clinic, with no formal appointments. Some women may not know the importance of attending ANC early, or may not know how far along in their pregnancy they should be before attending their first ANC appointment [5,9]. However, even with adequate knowledge, socio-cultural determinants of health-seeking behaviors may negatively impact utilization of ANC services [7].

In South Africa, all government health care facilities offer free basic antenatal care (BANC) services. While most women cannot afford the full cost of ANC and delivery in the private sector, some still seek initial ANC services in the private sector. When referred from the private to public sector care, these women are expected to carry letters or cards that summarise all relevant ANC up to that point. The government recommends that pregnant women attend their first ANC visit before 20 weeks gestation [11]; the rate of presentation for first ANC before 20 weeks is a core national indicator used to assess the performance of the national PMTCT programme. In 2015/16, 61.2% of pregnant women in South Africa attended their first ANC before 20 weeks gestation [12], and 94% attended at least one ANC visit to public sector healthcare facilities for that pregnancy [13]. However, 39 (75%) of South Africa’s 52 health districts achieved the 60% national target of initiating ANC before 20 weeks of pregnancy [12]. Critical to this context is the high prevalence of HIV in pregnant women, recently estimated at 29.5% [14]. Specifically, late presentation for ANC delays HIV diagnosis and ART initiation. Ultimately, delayed ART initiation decreases the length of time available for optimal viral load suppression prior to delivery, thus increasing the risk of mother-to-child transmission (MTCT) of HIV [15,16].

Despite the importance of presenting early for ANC services, few studies have been conducted in South Africa to determine risk factors for late first ANC presentation. As South Africa shifts its focus from prevention to elimination of MTCT of HIV, determining population-specific risk factors for late ANC presentation is crucial. Based on expressed concerns from two local health departments about their ANC attendance indicators, we sought to investigate risk factors for late presentation for first ANC visits in rural and peri-urban settings.

## Materials and methods

**Fig 1:**
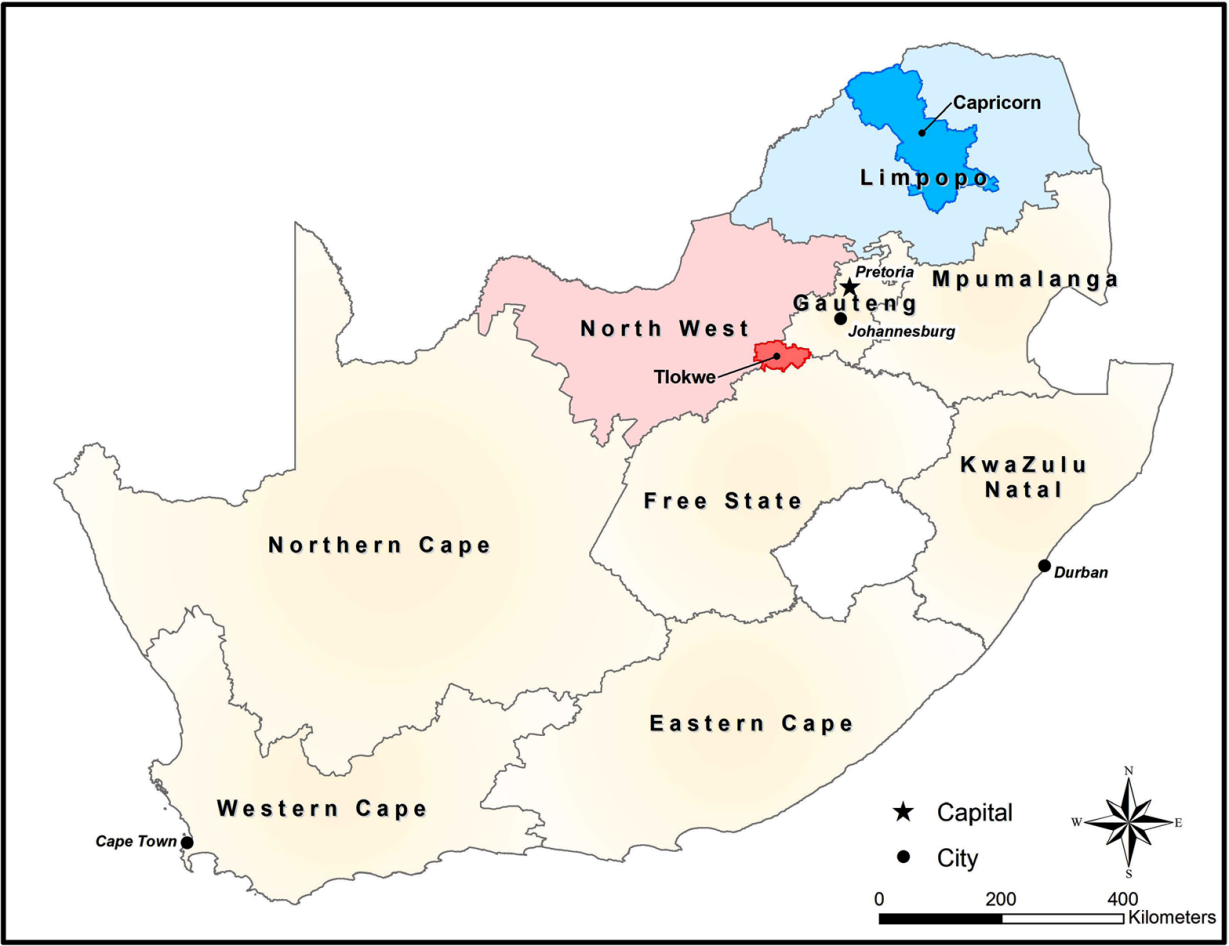
National map of South Africa with highlighted study locations.

A cross-sectional study was conducted amongst pregnant women attending primary healthcare (PHC) clinics in rural Capricorn District, Limpopo Province and peri-urban Tlokwe sub-district, Dr Kenneth Kaunda District, North West Province (Fig 1) for their first ANC visit of that pregnancy. The PHC is the foundational health facility in South Africa that “provides an integrated package of essential primary health care services available to the entire population”. It was implemented to promote equity and access to health care [17].

Both Capricorn and Tlokwe fell below the national target for first ANC visit before 20 weeks for 2013/14. The Foundation for Professional Development (FPD) was the PEPFAR District Support partner for Health Systems Strengthening activities in Capricorn District and Tlokwe sub-district. Health officials from both municipalities expressed concern about their rates of first ANC attendance before 20 weeks. As such, both locations were selected based on the districts seeking support to investigate these issues.

Capricorn is one of six districts in Limpopo Province, and has an estimated 2016 population of 1,330,463. Capricorn has 99 primary health clinics (PHCs) across five sub-districts. In 2013, an estimated 26,061 women in the district attended first ANC services across the clinics.

Tlokwe is a peri-urban sub-district in Dr Kenneth Kaunda District, North West Province, and has an estimated 2016 population of 179,604. The study was conducted in all eight primary healthcare clinics (PHCs) in Tlokwe sub-district. On average, the clinics in the sub-district attend to about 2,400 pregnant mothers coming for first antenatal visit per annum.

Study participants were recruited from 20 randomly selected PHCs in Capricorn and all eight PHCs in Tlokwe. Participant enrolment occurred from January 26^th^ through August 26^th^ 2015.

Sample sizes for each study location was determined using the South African District Health Information System’s average rate of first ANC presentation after 20 weeks (Capricorn = 43%; Tlokwe = 34%), with a 5% level of significance and 5% margin of error. The calculated sample size for Capricorn District was 377, adjusted by adding 5% non-response rate (n = 397) and then rounded off to 400. The sampling frame consisted of the 99 PHCs in Capricorn, stratified by sub-district. Clinic selection was based on probability proportional to the annual headcount of women presenting for first ANC at each sub-district. The calculated sample size for Tlokwe sub-district was 345, adjusted by adding 10% non-response rate, and rounded off to 397. This sample was proportionally allocated to Tlokwe’s eight PHCs based on their three-month average headcount of women presenting for first ANC. Consecutive consenting pregnant women were recruited in each clinic until the required sample size for that clinic was met. For Capricorn and Tlokwe, recruitment occurred over a variety of days and times each week. Some facilities offered ANC services every day while others only offered ANC on designated days of the week.

Trained study personnel conducted face-to-face interviews using a pre-piloted, structured questionnaire. Data was collected on socio-demographics, obstetric characteristics, and the knowledge, attitude and practice of the women with regards to ANC. Per South African national guidelines for ANC services, gestational age was measured using the date of last menstrual period of the mother and/or symphysis-fundal height; gestational ages were abstracted from maternity case records [18]. For the two areas, sampling occurred over a variety of days of the week and times. Some facilities offered ANC services every day while others offered it at a designated day of the week.

The South African National Department of Health defines late first ANC presentation as those women presenting to a government health clinic at or after 20 weeks gestation. As such, our outcome variable defines early and late presentation for first ANC as <20 weeks gestation vs. ≥20 weeks gestation, respectively. For the purpose of this study, we defined a ‘partner’ as the sexual partner for current pregnancy (i.e. the father of the baby).

Data were entered into a web-based data entry system and stored on an in-house server. Categorical data were summarized as proportions. Median, range and inter-quartile range (IQR) were used to describe numeric data. Pearson's chi-squared test or Fisher's exact test was used for comparison of categorical variables. Analysis was done, in the adolescent sub-population, to determine their effective use of family planning services. Logistic regression with fixed effect for Capricorn and Tlokwe (which would help establish if factors associated with ANC are different between areas more objectively) was used for both bivariate and multivariate analyses to assess factors associated with late presentation for first ANC. Variables with *P*<0.2 in bivariate analysis were entered into the multivariate model. A *P*<0.05 in the multivariate analysis was regarded as statistically significant and 95% confidence intervals (CI) were used to estimate precision. Goodness-of-fit test was used to compare observed values to what the model predicted. Data analysis was performed using STATA 13 (StataCorp LP, College Station, TX, USA).

Ethics approval was obtained from The Foundation for Professional Development Research Ethics Committee (REC-03711-033-RA). Study approvals were obtained from Capricorn District and Limpopo Provincial health departments, and the Tlokwe sub-district and North West Provincial health departments. Written informed consent was obtained from all participants prior to data collection.

## Results

A total of 810 pregnant women attending their first ANC visit for that pregnancy were recruited from Capricorn District (n = 410) and Tlokwe sub-district (n = 400; Table 1); three women recruited from Tlokwe were excluded from all analysis due to missing information on gestational age. The age of the study participants ranged from 14 to 44 years, with most (58%) within the 20–29 years.

**Table 1:** Socio-demographic, obstetric and health seeking characteristics of study participants.

	Total	Capricorn	Tlokwe	
Variables	n = 807	n = 410	n = 397	*P-value*
**Maternal age group**				*0*. *981*
<20 Years	110 (14)	56 (14)	54 (14)	
20–29 Years	468 (58)	229 (56)	239 (60)	
30–39 Years	202 (25)	111 (27)	91 (23)	
40+ Years	27 (3)	14 (3)	13 (3)	
**Marital status**				***0*.*036***
Unmarried^a^	677 (84)	333 (81)	344 (87)	
Married	130 (16)	77 (19)	53 (13)	
**Employed**				***<0*.*001***
Yes	214 (27)	76 (19)	138 (35)	
No	593 (73)	334 (81)	259 (65)	
**Partner employed**	(n = 799)	(n = 402)	(n = 396)	***<0*.*001***
Yes	567 (71)	249 (62)	318 (80)	
No	232 (29)	153 (38)	79 (20)	
**Education level**				***<0*.*001***
Tertiary	130 (16.1)	103 (25.1)	27 (6.8)	
Matric	287 (35.6)	136 (33.2)	151 (38)	
Less than matric	383 (47.5)	165 (40.2)	218 (54.9)	
None	7 (0.8)	6 (1.5)	1 (0.3)	
**Monthly household income**	(n = 792)	(n = 395)	(n = 397)	***<0*.*001***
None	48 (6)	41 (10.4)	7 (2)	
<R1000	120 (15)	65 (16.4)	55 (14)	
R1001-R4000	381 (48)	181 (45.8)	200 (51)	
R4001-R8000	117 (15)	52 (13.2)	65 (16)	
>R8000	126 (16)	56(14.2)	70 (17)	
**Alcohol use**				*0*. *114*
Yes	78 (10)	33 (8)	45 (11)	
**Drug use**				*0*. *325*
Yes	1 (0. 1)	1 (0. 2)	0 (0)	
**Tobacco use**				***<0*.*001***
Yes	66 (8)	17 (4)	49 (12)	
**Gravidity**				*0*. *743*
Multigravida	533 (66)	273 (67)	260 (65)	
Primigravida	274 (34)	137 (33)	137 (35)	
**Parity**				*0*. *384*
Para one and above	492 (61)	256 (62)	236 (59)	
Nulliparous	315 (39)	154 (38)	161 (41)	
**Previous ANC use**	(n = 513)	(n = 261)	(n = 252)	*0*.*7*
Yes	474 (92)	240 (92)	234 (93)	
No	39 (8)	21 (8)	18 (7)	
**Actual timing of 1st ANC visit**				***<0*.*001***
<20 weeks (Early)	488 (60)	202 (49)	286 (72)	
≥20 weeks (Late)	319 (40)	208 (51)	111 (28)	
**Trimester at 1st ANC presentation**				***<0*.*001***
1st (0–12 weeks)	222 (28)	71 (17.3)	151 (38)	
2nd (13–27 weeks)	493 (61)	276 (67.3)	217 (54.7)	
3rd (28+ weeks)	92 (11)	63 (15.4)	29 (7.3)	
**Unplanned Pregnancy**				*0*.*134*
Yes	487 (60)	237 (58)	250 (63)	
No	320 (40)	173 (42)	147 (37)	
**Reported barriers to accessing ANC services**				***<0*.*001***
Yes	227 (28)	176 (43)	51 (13)	
No	580 (72)	234 (57)	346 (87)	
**Experienced booking delay**				***<0*.*001***
Yes	192 (24)	31 (8)	161(41)	
No	615 (76)	379 (92)	236 (59)	
**Median (IQR) booking delay in days**	3 (1–5)	6 (1–7)	2 (1–5)	
**First care on confirmation of pregnancy**				***0*.*001***
Outside Public Systems	139 (17)	89 (22)	50 (13)	
Public Clinic	668 (83)	321 (78)	347 (87)	
**Reasons for care outside public health system**	(n = 139)	(n = 89)	(n = 50)	***<0*.*001***
Better care	47 (33. 8)	36 (41)	11 (22)	
More convenient	53 (38. 2)	23(26)	30 (60)	
Short waiting time	20 (14. 4)	11 (12)	9 (18)	
Someone else told me to	18 (12. 9)	18 (20)	0 (0)	
Prevent witchcraft	1 (0. 7)	1 (1)	0 (0)	
**Gestational age at 1st ANC vi in public sector after presenting early at private medical care**	(n = 115)	(n = 80)	(n = 35)	***0*.*002***
<20 weeks (Early)	71 (62)	42(53)	29 (83)	
≥20 weeks (Late)	44(38)	38(47)	6 (17)	
**Basic ANC services received at private medical care**	(n = 139)	(n = 89)	(n = 50)	
Pregnancy test	104 (75)	64 (72)	40 (80)	
HIV test	32 (23)	17 (19)	15 (30)	
Blood pressure check	78 (56)	41 (46)	37 (74)	
Iron pills	77 (55)	40 (45)	37 (74)	
Physical examination	71 (51)	38 (43)	33 (66)	
Syphilis test	8 (5. 8)	4 (5)	4 (8)	
Vaccination	9 (6. 5)	3 (3)	6 (12)	
Education on pregnancy	36 (26)	22 (25)	14 (28)	
Other	22 (16)	17(19)	5 (10)	

Data are n(%) unless otherwise specified.

Bold indicates statistical significance of *P*<0.05.

R, South African Rand

^a^Single, divorced, co-habiting or widowed

IQR, Interquartile Range; ANC, antenatal clinic.

Though no significant differences in obstetric characteristics was detected between the study populations from Capricorn and Tlokwe, the two differed significantly in certain socio-demographic characteristics and attitudes towards ANC. Specifically, a higher proportion of participants in Capricorn reported they were married (19% vs. 13%; p 0.036); unemployed (81% vs. 65%; p <0.001); have partners that are unemployed (38% vs. 20%; p <0.001); have higher levels of education (58% vs. 45%; p <0.001) and have no monthly income (10.4% vs. 2%; p <0.0001). Conversely, a higher proportion of participants in Tlokwe reported using tobacco products (12% vs. 4%; p <0.001).

Of the 807 study participants, 319 (40%) presented late (≥ 20 weeks) for their first ANC, with about 11% within the third trimester. Participants in Capricorn were more likely to present late than those in Tlokwe (51% vs. 28%; p <0.001) and also within the third trimester (15.4% vs. 7.3%; p <0.001). Of note, early ANC is considered by WHO to be up to 12 weeks gestation [19]. By WHO standards, 72% (585/807) of women in our study presented late for their first ANC visit (Capricorn: 83% (339/410); Tlokwe: 62% (246/397). Gravidity, parity, and number of previous pregnancies did not vary between Capricorn and Tlokwe (Table 1). More than half of all participants reported that their pregnancy was unplanned (Table 1), with more adolescents (<20 years) in both settings reporting an unplanned pregnancy compared to women 20 years or more (82% vs. 57%; p <0.001). First ANC delays due to clinic bookings, where women present early to the clinics but are given an appointment to initiate ANC at a later date, was reported more by participants from Tlokwe (41% vs. 8%; p <0.001).

Once pregnancy was confirmed, 22% of participants in Capricorn and 13% in Tlokwe reported seeking care from non-public sector health care providers (e.g., general practitioners, consultant gynecologists or traditional healers; *P* = 0.001; Table 1). Reasons for seeking care outside of the public health system varied significantly by study setting (Table 1; *P*<0.001). In both Tlokwe and Capricorn, no participant could recall receiving the full package of ANC services from non-public sector health care providers; fewer than 25% of participant receiving an HIV test in the non-public health sector (Table 1). Furthermore, 38% (44/115) of those that first sought care outside the public sector presented late for first ANC at public sector clinics.

**Fig 2:**
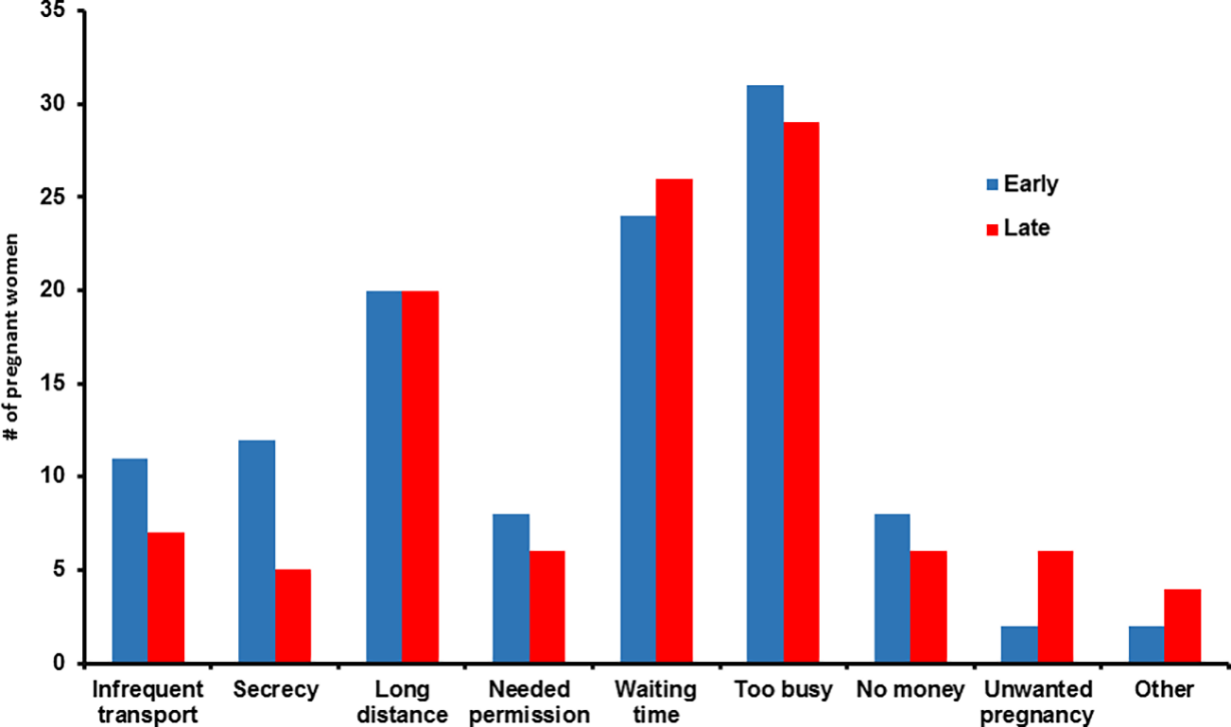
Difficulties with coming to the clinic for ANC and first ANC entry time (n = 227).

Participants in Capricorn were significantly more likely to report a barrier to accessing ANC services than those in Tlokwe (43% vs. 13%; p <0.001). Of those reporting barriers to accessing ANC services (n = 227; Fig 2) the top five reported barriers were 1) being too busy (n = 60), 2) long waiting time (n = 50), 3) distance to the clinic (n = 40), 4) infrequent transport (n = 18) and 5) cultural secrecy (n = 17). Of the 30 women who reported being too busy and presented late for their first ANC, 17 (57%) were unemployed. After adjusting for study location, there was no significant association found between reporting any barrier to accessing care and presenting late for first ANC services.

An assessment of participant’s knowledge, perceptions and practices relating to ANC (Table 2) revealed that of the 546 (68%) study participants who indicated knowing the right gestational age at which a pregnant woman should attend their first ANC visit, 506 (93%) correctly reported start time of before 20 weeks’ gestation (Table 2). Of those that correctly reported the recommended ANC starting time, 38% (194/506) presented late for their first ANC visit. Nearly half of all participants reported receiving information or advice of when to start ANC, with the majority receiving information from health workers (Table 2). Very few women were accompanied to the clinic by their partner (7%), and ~30% of women expressed a negative emotion (e.g. sad or scared) upon confirmation of their pregnancy.

**Table 2:** ANC knowledge, attitudes and practices of study participants.

	Total	Capricorn	Tlokwe	
Variables	n = 807	n = 410	n = 397	*P-value*
**Have knowledge on right time to start ANC**				***<0*.*001***
Yes	546 (68)	328 (80)	218 (55)	
No	261 (32)	82 (20)	179 (45)	
**Of those with knowledge, timing of 1st ANC**	(n = 546)	(n = 328)	(n = 218)	*0*.*319*
<20 weeks (Early)	506 (93)	301 (92)	205 (94)	
≥20 weeks (Late)	40 (7)	27 (8)	13 (6)	
**Received information/advise on when to start ANC**				*0*. *632*
Yes	385 (48)	199 (49)	186 (47)	
No	422 (52)	211 (51)	211 (53)	
**Source of information on starting ANC**	(n = 385)	(n = 199)	(n = 186)	*0*.*088*
Friends	55 (14)	2 (11)	33 (18)	
Health workers	201 (52)	113 (57)	88 (47)	
Media	13 (4)	4 (2)	9 (5)	
Relatives	109 (28)	55 (28)	54 (29)	
Other	7 (2)	5 (2)	2 (1)	
**Reason for coming to the clinic**				***0*.*002***
It is time to start ANC	572 (71)	278 (68)	294 (74)	
Known risk factor (Age, Status, History)	55 (7)	36 (9)	19 (5)	
Sickness	96(12)	40 (10)	56 (14)	
Told to come by someone else	76 (9)	51 (12)	25 (6)	
Other	8 (1)	5 (1)	3 (1)	
**Accompanied to the clinic by partner**				*0*. *556*
Yes	53 (7)	29 (7)	24 (6)	
No	754 (93)	381 (93)	373(94)	
**Feeling when pregnancy was confirmed**				***<0*.*001***
Happy	401 (49.7)	183 (45)	218 (55)	
Mixed emotions	14 (1.7)	11 (3)	3 (1)	
Sad	124 (15.4)	63 (15)	61 (15)	
Scared	122 (15.1)	55 (13)	67 (17)	
Surprised	146 (18.1)	98 (24)	48 (12)	

IQR, Interquartile range; ANC, antenatal clinic.

Data are n(%) unless otherwise specified.

Bold indicates statistical significance of *P*<0.05.

The goodness-of-fit test shows our model fits adequately well with the observed outcome in the data (observations 807; prob>chi2 0.4941). The rural women were more likely to present late for first ANC (AOR = 2.65; 95% CI 1.98–3.55; p = <0.001). Factors associated with late first ANC presentation (Table 3) in Capricorn are 1) 20–29 years of age (AOR = 0.42; 95% CI 0.21–0.84), 2) married (AOR = 1.97; 95% CI 1.12–3.48), and 3) reported an unplanned pregnancy (AOR = 2.10; 95% CI 1.35–3.27). Alternately, in Tlokwe, significant associations exist between late presentation for first ANC and women who reported an unplanned pregnancy (AOR = 1.80; 95% CI 1.09–2.96). Those who were pregnant for the first time (AOR = 0.55; 95% CI 0.33–0.91), told to come back later for ANC initiation even after presenting early to the clinic (AOR 0.61; 95% CI 0.37–0.98) and those who received information or advice on when to start ANC (AOR 0.61; 95% CI 0.38–0.98) were less likely to initiate ANC late (Table 3). The results of the pooled multivariate analysis showed that older age group has lower odds of initiating ANC late compared to those below 20 years (adolescents), as well as those who received information or advice on when to start ANC (AOR 0.70; 95% CI 0.52–0.96). The women who reported unplanned pregnancy (AOR 1.97; 95% CI 1.43–2.72) were more likely to initiate ANC late (Table 4).

**Table 3:** Logistic regression analysis (with fixed effects) of determinants of late presentation in rural Capricorn District and peri-urban Tlokwe sub-district.

	CAPRICORN						TLOKWE					
	Time at 1^st^ ANC		Bivariate Analysis		Multivariate Analysis		Time at 1^st^ ANC		Bivariate Analysis		Multivariate Analysis	
	Early	Late	OR (95% CI)	*P*	AOR (95% CI)	*P*	Early	Late	OR (95% CI)	*P*	AOR (95% CI)	*P*
Variables	202 (49)	208 (51)					286 (72)	111 (28)				
**Maternal age group**												
<20 Years	18 (32)	38 (68)	Ref		Ref		37 (69)	17 (31)	Ref			
20–29 Years	128 (56)	101 (44)	0.37 (0.20–0.69)	**0.002**	0.42 (0.21–0.84)	**0.014**	181 (76)	58 (24)	0.69 (0.37–1.33)	0.275		
30–39 Years	53 (48)	58 (52)	0.52 (0.27–1.02)	**0.056**	0.49 (0.23–1.05)	0.068	59 (65)	32 (35)	1.18 (0.58–2.42)	0.651		
40+ Years	3 (21)	11 (79)	1.73 (0.43–6.99)	0.438	1.12 (0.26–4.78)	0.88	9 (69)	4 (31)	0.97 (0.26–3.58)	0.96		
**Marital status**												
Unmarried	171 (85)	162 (78)	Ref		Ref		248 (87)	96 (86)	Ref			
Married	31 (15)	46 (22)	1. 56 (0.95–2.59)	**0.081**	1.97 (1.12–3.48)	**0.019**	38 (13)	15 (14)	1.02 (0.54–1.94)	0.953		
**Employed**												
Yes	33 (16)	43 (21)	1. 33 (0.81–2.20)	**0.26**	1.60 (0.91–2.80)	0.103	105 (37)	33 (30)	0. 73 (0.46–1.17)	**0.191**	0.68 (0.41–1.11)	0. 121
**Partner employed**	(n = 402)						(n = 396)					
Yes	131 (65.5)	118 (58.4)	0. 74 (0.49–1.11)	**0.145**	0.85 (0.54–1.36)	0.504	228 (80)	90 (81)	1.09 (0.63–1.90)	0.761		
**Education level**												
Tertiary	59 (57)	44 (43)	Ref		Ref		18 (67)	9 (33)	Ref			
Matric	71 (52)	65(48)	1.23 (0.73–2.05)	0.436	1.07 (0.62–1.85)	0.813	113 (75)	38 (25)	0.67 (0.28–1.62)	0.453		
Less than matric	68 (41)	97 (59)	1.91 (1.16–3.14)	**0.011**	1.42 (0.82–2.45)	0.213	154 (71)	64 (29)	0.83 (0.36–1.95)	0.671		
None	4 (67)	2 (33)	0.67 (0.12–3.82)	0.653	0.40 (0.06–2.51)	0.322	1 (100)	0 (0)	-	-		
**Gravidity**												
Primigravida	70 (35)	67 (32)	0.89 (0.59–1.39)	0.601			106 (37)	31 (28)	0.66 (0.40–1.06)	**0.087**	0.55 (0.33–0.91)	**0.021**
Multigravida	132 (65)	141 (68)	Ref				180 (63)	80 (72)	Ref			
**Previous ANC use**	(n = 261)						(n = 252)					
Yes	116 (93)	124 (91)	0. 80 (0.33–1.97)	0.631			161 (93)	73 (92)	0.91 (0.33–2.51)	0.851		
**Unplanned pregnancy**												
Yes	97 (48)	140 (67)	2.22 (1.49–3.12)	**<0.001**	2.10 (1.35–3.27)	**0.001**	170 (59)	80 (72)	1.76 (1.09–2.83)	**0.02**	1.80 (1.09–2.96)	**0.021**
**Received information/ advice on when to start ANC**												
Yes	105 (52)	94 (45)	0. 76 (0.52–1.12)	**0.17**	0.74 (0.49–1.12)	0.152	145 (51)	41 (37)	0.57 (0.36–0.89)	**0.014**	0.61 (0.38–0.98)	**0.04**
												
**Have knowledge on right time to start ANC**												
Yes	165 (82)	163 (78)	0. 81 (0.50–1.32)	0.402			163 (57)	55 (49.5)	0.74 (0.48–1.15)	**0.182**	0.77 (0.48–1.24)	0.287
												
**Experienced booking delay**												
Yes	14 (7)	17 (8)	1. 19 (0.57–2.49)	0.635			127 (44)	34 (31)	0.55 (0.35–0.88)	**0.013**	0.61 (0.37–0.98)	**0.042**
												
**Reported barriers to accessing ANC services**												
Yes	80 (40)	96 (46)	1. 31 (0.88–1.93)	**0.181**	1.20 (0.79–1.82)	0.403	38 (13)	13 (12)	0.87 (0.44–1.69)	0.674		
												
**First medical care on confirmation of pregnancy**												
Private	46 (23)	43 (21)	0. 88 (0.55–1.41)	0.607			32 (11)	18 (16)	1.53 (0.82–2.86)	**0.178**	2.03 (1.02–4.06)	**0.044**
Public Clinic	156 (77)	165 (79)	Ref				254 (89)	93 (84)	Ref			

OR, Odds Ratio; AOR, Adjusted Odds Ratio; CI, Confidence Interval; ANC, antenatal clinic.

Data are n (%) unless otherwise specified.

As stated in the Materials and Methods, bivariate variables with *P*>0.2 were excluded from multivariate models.

Bold indicates statistical significance of *P*<0.05.

**Table 4:** Pooled logistic regression analysis (with fixed effects) of determinants of late presentation.

	Time at 1^st^ ANC Entry		Bivariate Analysis		Multivariate Analysis	
	Early (<20 weeks)	Late (≥20 weeks)	OR (95% CI)	*P*	AOR (95% CI)	*P*
Variables	n = 488 (60)	n = 319 (40)				
**Maternal age group**						
<20 Years	55 (50)	55 (50)	Ref		Ref	
20–29 Years	309 (66)	159 (34)	0.51 (0.33–0.78)	**0.002**	0.51 (0.31–0.84)	**0.008**
30–39 Years	112 (55)	90 (45)	0.76 (0.47–1.23)	**0.266**	0.64 (0.35–1.16)	0.141
40+ Years	12 (44)	15 (56)	1.25 (0.53–2.99)	0.61	0.97 (0.37–2.55)	0.953
**Marital status**						
Married	69 (14)	61 (19)	1.33 (0.90–1.95)	**0.124**	1.31 (0.85–1.99)	0.219
Unmarried	419 (86)	258 (81)	Ref		Ref	
**Employed**						
Yes	138 (28)	76 (24)	0.96 (0.69–1.36)	0.844		
**Partner employed** (n = 799)						
Yes	359 (74)	208 (66)	0.85 (0.61–1.17)	0.319		
**Education level**						
Tertiary	77 (59)	53 (41)	Ref		Ref	
Matric	184 (64)	103 (36)	1.12 (0.72–1.75)	0.608	0.92 (0.58–1.47)	0.737
Less than matric	222 (58)	161 (42)	1.55 (1.01–2.38)	**0.044**	1.15 (0.72–1.84)	0.546
None	5 (71)	2 (29)	0.54 (0.01–2.93)	0.475	0.48 (0.08–2.93)	0.431
**Gravidity**						
Primigravida	176 (36)	98 (31)	0.79 (0.58–1.07)	**0.126**	0.71 (0.48–1.04)	0.081
Multigravida	312 (64)	221 (69)	Ref		Ref	
**Previous ANC use** (n = 513)						
Yes	227 (93)	197 (92)	0.85 (0.43–1.65)	0.627		
**Unplanned pregnancy**						
Yes	267 (55)	220 (69)	2.02 (1.49–2.74)	**<0.001**	1.97 (1.43–2.72)	**<0.001**
**Received information/ advice on when to start ANC**						
Yes	250 (51)	135 (42)	0.67 (0.50–0.90)	**0.008**	0.70 (0.52–0.96)	**0.025**
**Have knowledge on right time to start ANC**						
Yes	328 (67)	218 (68)	0.77 (0.56–1.07)	0.121	0.90 (0.64–1.27)	0.555
**Experienced booking delay**						
Yes	141 (29)	51 (16)	0.69 (0.47–1.01)	**0.059**	0.67 (0.45–1.01)	0.054
**Reported barriers to accessing ANC services**						
Yes	118 (24)	109 (34)	1.17 (0.84–1.64)	0.351		
**First medical care on confirmation of pregnancy**						
Private	78 (16)	61 (19)	1.07 (0.73–1.57)	0.715		
Public Clinic	410 (84)	258 (81)	Ref			

OR, Odds Ratio; AOR, Adjusted Odds Ratio; CI, Confidence Interval; ANC, antenatal clinic.

Data are n(%) unless otherwise specified.

As stated in the Materials and Methods, bivariate variables with *P*>0.2 were excluded from multivariate models.

Bold indicates statistical significance of *P*<0.05.

## Discussion

Though South Africa considers women presenting for their first ANC visit before 20 weeks gestation as presenting early for ANC, a large proportion (72%) of our study population presented for their first ANC beyond the WHO recommendation for initiating antenatal care within the first trimester. The implication of presenting late for ANC is the delayed opportunity to prevent adverse pregnancy outcomes, for both mother and child. We found that pregnant women living in the rural communities were more likely to present late for their first ANC visit than those living in the peri-urban communities This finding is consistent with previous studies in Ethiopia [5,20]. Furthermore, being married or <20 years of age were risk factors unique to our rural community, while booking delays, being pregnant for the first time and receiving information or advice on when to start ANC were unique risk factors to our peri-urban community. Having an unplanned pregnancy was a common risk factor to both communities. Our finding that rural women were more likely to present late for their first ANC compared to peri-urban women may be attributable to differences in certain socio-demographic characteristics and attitudes towards ANC between the rural and peri-urban population in our study.

In our study, unplanned pregnancy was an independent risk factor for late ANC presentation in both study communities. Those with an unplanned pregnancy may be missing familial or partner support that engenders good healthcare seeking behavior [21]. Furthermore, women with unplanned pregnancies may contemplate pregnancy termination [22] or may be in denial of the pregnancy [23] both of which may delay presentation for ANC services. Unplanned pregnancies are also related to social-cultural determinants of health-seeking behaviours, sexual violence and barriers to access; some of these may be associated with late ANC. For instance, barriers to access family planning may be similar to those to access ANC. Effective use of family planning services should be further promoted to reduce events of unintended pregnancies and late ANC presentations [22]. Increased effort should also be made to improve women’s behaviour towards ANC use, especially those with unplanned pregnancies.

Adolescent girls (15–19 years) account for more than 10% of births world-wide and are more likely than older mothers to die in child birth [24]. Our findings that adolescent girls were more likely to present late for their first ANC visit and have unplanned pregnancies are consistent with previous findings [24,25]. Given the social consequences of pregnancy during adolescence, factors that influence ANC attendance among adolescents in these settings require further studies.

Knowledge about the timing of first ANC was associated with presentation for ANC in the combined data. The pregnant women who received information or advice on when to start ANC were less likely to start ANC late. This is consistent with findings from two Ethiopian studies [26,27] but differed from a Tanzanian study that found no association between knowledge about correct ANC timing and early ANC presentation [28]. Our study also observed a tendency for late ANC initiation among women who had been pregnant more than once. This is in line with our finding that previous pregnancies did not offer protection against late ANC entry. This could mean the women were not adequately educated through ANC about the importance of presenting on time for the next pregnancy or they feel starting early is not necessary if there were no complications with the previous pregnancy. Some women may feel more confident after previous birth experiences and delay ANC initiation [3] and difficulty in finding someone to look after their children may also explain the same finding in other settings. In our study, 48% of study participants indicated they received information on when to start ANC mainly from healthcare workers. Considering the substantial number of primigravida women (34%) in our study, community-based sources of information on when to start ANC are needed to encourage early presentation.

The marital status of the rural women was found to be significantly associated with late ANC presentation. Specifically, married women were more likely to initiate ANC late than unmarried women. This finding is contrary to observations in Rwanda, where being married was protective against late ANC entry [6], and Tanzania, where no significant association was identified [28]. We also observed that being accompanied to the clinic for ANC by their partners did not seem to be the usual practice as only 7% (53/807) of the pregnant women were accompanied to the clinic by their partners.

South African guidelines on maternity care require ANC services be provided during first contact with a pregnant woman, and without formal appointments. Interestingly, we found that booking delays (i.e., being told to return at a later date to initiate first ANC visit services) in Tlokwe sub-district were associated with early first ANC presentation. It is not clear why this is the case, as booking delays have previously been reported as a barrier to early ANC presentation [10].

We did not consider presentation for first ANC before 20 weeks to a non-public sector health provider as an early visit because participants did not have any documentation on their visit, nor did any report receiving the comprehensive set of ANC services. Our findings of women presenting early (i.e., before 20 weeks gestation) to non-public sector providers, but then presenting late to public sector clinics is consistent with findings by Sibeko and Moodley in Durban, South Africa [2]. It would appear that pregnant women confirm their pregnancies by visiting general practitioners, and then present for ANC services in the public sector. According to the guidelines for maternal care in South Africa, complete assessment of gestational age and risk factors ought to be made at the first antenatal visit. Although the capacity to provide comprehensive ANC services in the non-public sector is available in South Africa, most women cannot afford the same set of comprehensive services that the public sector provides in the private sector. It is tempting to suggest that South Africa’s programmatic definition may result in an overestimation of the rate of late first ANC presentation. However, the incomplete package of basic antenatal care services provided by non-public sector health care providers, especially the low rates of HIV testing, suggests that caution should be taken in interpreting these results, especially in the context of risk reduction services for prevention of mother-to-child transmission of HIV.

The tendency of the employed women in our rural community to present late for their first ANC visit is in agreement with a—recent study in Johannesburg [10]. However, we observed that 17 of 30 57%) women in our total population who presented late because of their busy schedule were unemployed. This suggests that in these communities, employment is not the only measure or explanation for a busy schedule. Thus, understanding the daily lives of women in these communities is needed to better inform interventions to assure early presentation for ANC services.

Study participants from Capricorn were more educated but less likely to be employed or have monthly income. This should not logically be the case. It is possible the women reported being unemployed to avoid any hospital charges at the time of delivery. Although ANC services are offered free at all levels of care in South Africa, not all of them have obstetric units. Delivery is mainly done in community health centres and hospitals. The patients are expected to pay a minimal service fee, the amount depending on annual income to ensure those who can pay do so.

Our study had some limitations. The study only included women attending ANC at public health clinics. As a cross-sectional study, the associations observed may not be causal enough. Specifically, the list of risk factors we measured was likely not comprehensive, and factors relating to the health care systems or the attitude of healthcare workers were excluded. Including qualitative in-depth interviews may have enhanced our understanding of the reasons for women presenting late for their first ANC visit. Our data is not representative of all women, but only those who sought antenatal care at public facilities. Participants not being randomly selected is also a weakness of the study. There was a sampling frame for selecting the clinics per sub-district but not for individuals within the clinics. Quality of care differences between the public and the non-public sectors was not assessed in the study. The participants were interviewed before they received their basic ANC screenings in the public health facilities and not again after.

The observed late ANC attendance in our participants indicates that the importance of early ANC is not yet generally understood by pregnant women despite high ANC coverage in South Africa. As such, we recommend conducting research to identify optimal messaging and modes of communication to improve early presentation and increase demand generation for ANC. From a public health perspective, ANC received from a skilled provider is desirable, irrespective of the type of provider. It should be emphasized that the concerns surrounding seeking ANC services outside the government health systems is not one of a women’s behaviour or understanding of the need for ANC, but that of the quality of services in the private sector. However, complete package of ANC services at the public health facilities is not always guaranteed, as some services, like HIV testing, may not be available at a woman’s first ANC visit, even when it is required. Given the high prevalence of HIV in pregnant women in South Africa, we generally recommend all health providers provide the full BANC, especially general practitioners who can facilitate early referral to public ANC clinics when full BANC cannot be provided. Although South Africa is currently implementing a community health worker (CHW) program aimed at decentralizing primary health services to the community level, we have no data to suggest that CHW activities in these two districts may have differential impact on timing of first ANC attendance; this area requires future study.

The prevalence of late ANC presentation was significantly higher in the rural compared to the peri-urban study area. The predictors of presenting late for first ANC varied between rural and peri-urban study populations. Our findings highlight the need for community-based health education on the importance of early ANC initiation to empower women with this information before they get pregnant. Interventions to reduce prevalence of late ANC presentation should also address unplanned pregnancy through family planning education, barriers to access of healthcare services and social determinants of health-seeking behaviour. Since pregnant women are more likely to visit private GPs to confirm their pregnancies, the capacity of GPs to provide appropriate ANC services should be strengthened.

## Supporting information

S1 File Combined dataset for NW_LP ANC study. (DTA)

## References

[1] Lawn JE, Lee AC, Kinney M, Sibley L, Carlo WA, Paul VK, et al Two million intrapartum-related stillbirths and neonatal deaths: Where, why, and what can be done? Int J Gynecol Obstet. 2009 10;107:S5–19. 10.1016/j.ijgo.2009.07.016 19815202

[2] Sibeko S, Moodley J. Healthcare attendance patterns by pregnant women in Durban, South Africa. South African Fam Pract. 2006 11;48(10):17–17e.

[3] Banda I, Michelo C, Hazemba A. Factors associated with late antenatal care attendance in selected rural and urban communities of the copperbelt province of Zambia. Med J Zambia. 2014;39(3):29–36.

[4] Simkhada B, Teijlingen ER van, Porter M, Simkhada P. Factors affecting the utilization of antenatal care in developing countries: systematic review of the literature: Factors affecting the utilization of antenatal care. J Adv Nurs. 2008 1;61(3):244–60. doi: 10.1111/j.1365-2648.2007.04532.x 18197860 10.1111/j.1365-2648.2007.04532.x

[5] Gudayu TW, Woldeyohannes SM, Abdo AA. Timing and factors associated with first antenatal care booking among pregnant mothers in Gondar Town; North West Ethiopia. BMC Pregnancy Childbirth. 2014 12;14(1). 10.1186/1471-2393-14-287 PMC4152591 25154737

[6] Manzi A, Munyaneza F, Mujawase F, Banamwana L, Sayinzoga F, Thomson DR, et al Assessing predictors of delayed antenatal care visits in Rwanda: a secondary analysis of Rwanda demographic and health survey 2010. BMC Pregnancy Childbirth. 2014 12;14(1). 10.1186/1471-2393-14-290 PMC4152595 25163525

[7] Ndidi E, Oseremen I. Reasons given by pregnant women for late initiation of antenatal care in the Niger Delta, Nigeria. Ghana Med J. 2011 8;44(2). PMC2994152 21327003

[8] Myer L, Harrison A. Why do women seek antenatal care late? Perspectives from rural South Africa. J Midwifery Womens Health. 2003 7;48(4):268–72. 12867911 10.1016/s1526-9523(02)00421-x

[9] Kisuule I, Kaye DK, Najjuka F, Ssematimba SK, Arinda A, Nakitende G, et al Timing and reasons for coming late for the first antenatal care visit by pregnant women at Mulago hospital, Kampala Uganda. BMC Pregnancy Childbirth. 2013 12;13(1). 10.1186/1471-2393-13-121 PMC3665546 23706142

[10] Solarin I, Black V. “They told me to come back”: women’s antenatal care booking experience in inner-city Johannesburg. Matern Child Health J. 2013 2;17(2):359–67. doi: 10.1007/s10995-012-1019-6 22527767 10.1007/s10995-012-1019-6 PMC3587683

[11] Pattinson RC. Basic antenatal care handbook Pretoria Univ Pretoria 2007;

[12] Massyn N, Day C, Peer N, Padarath A, Barron P, Day C, et al District Health Barometer 2015/2016. Durb Health Syst Trust 2016;

[13] Statistics South Africa. South Africa Demographic and Health Survey 2016: Key Indicator Report. 2016.

[14] National Department of Health. The 2013 National Antenatal Sentinel HIV Prevalence Survey South Africa. Health Systems Trust Available from: http://www.hst.org.za/publications/2013-national-antenatal-sentinel-hiv-prevalence-survey-south-africa

[15] Hoffman RM, Black V, Technau K, van der Merwe KJ, Currier J, Coovadia A, et al Effects of highly active antiretroviral therapy duration and regimen on risk for mother-to-child transmission of HIV in Johannesburg, South Africa. J Acquir Immune Defic Syndr 1999. 2010 5 1;54(1):35–41. 10.1097/QAI.0b013e3181cf9979 PMC2880466 20216425

[16] Black V, Hoffman RM, Sugar CA, Menon P, Venter F, Currier JS, et al Safety and efficacy of initiating highly active antiretroviral therapy in an integrated antenatal and HIV clinic in Johannesburg, South Africa. J Acquir Immune Defic Syndr 1999. 2008 11 1;49(3):276–81. 10.1097/QAI.0b013e318189a769 PMC2893046 18845949

[17] The Primary Health Care Package for SA—A Set of Norms and Standards. Western Cape Government. Available from: https://www.westerncape.gov.za/general-publication/primary-health-care-package-sa-set-norms-and-standards

[18] National Department of Health. Guidelines for Maternity Care in South Africa: A Manual for Clinics, Community Health Care Centres and District Hospitals. Pretoria: National Department of Health; 2015. Report No.: 4th edition.

[19] World Health Organization. WHO recommendations on antenatal care for a positive pregnancy experience WHO Available from: http://www.who.int/reproductivehealth/publications/maternal_perinatal_health/anc-positive-pregnancy-experience/en/28079998

[20] Gebremeskel F, Dibaba Y, Admassu B. Timing of first antenatal care attendance and associated factors among pregnant women in Arba Minch Town and Arba Minch District, Gamo Gofa Zone, south Ethiopia. J Environ Public Health. 2015;971506. 10.1155/2015/971506 PMC4620253 26543485

[21] Exavery A, Kanté AM, Hingora A, Mbaruku G, Pemba S, Phillips JF. How mistimed and unwanted pregnancies affect timing of antenatal care initiation in three districts in Tanzania. BMC Pregnancy Childbirth. 2013;13:35 doi: 10.1186/1471-2393-13-35 23388110 10.1186/1471-2393-13-35 PMC3574825

[22] Haddad DN, Makin JD, Pattinson RC, Forsyth BW. Barriers to early prenatal care in South Africa. Int J Gynaecol Obstet Off Organ Int Fed Gynaecol Obstet. 2016 1;132(1):64–7. 10.1016/j.ijgo.2015.06.041 26439856

[23] Kost K, Landry DJ, Darroch JE. Predicting maternal behaviors during pregnancy: does intention status matter? Fam Plann Perspect. 1998 4;30(2):79–88. 9561873

[24] McIntyre P, World Health Organization. Pregnant adolescents: delivering on global promises of hope Geneva: World Health Organization; 2006.

[25] Pell C, Meñaca A, Were F, Afrah NA, Chatio S, Manda-Taylor L, et al Factors affecting antenatal care attendance: results from qualitative studies in Ghana, Kenya and Malawi. PloS One. 2013;8(1):e53747 doi: 10.1371/journal.pone.0053747 23335973 10.1371/journal.pone.0053747 PMC3546008

[26] Zegeye AM, Bitew BD, Koye DN. Prevalence and determinants of early antenatal care visit among pregnant women attending antenatal care in Debre Berhan Health Institutions, Central Ethiopia. Afr J Reprod Health. 2013 12;17(4):130–6. 24558789

[27] Gudayu TW. Proportion and Factors Associated with late Antenatal Care Booking among Pregnant Mothers in Gondar Town, North West Ethiopia. Afr J Reprod Health. 2015 6;19(2):94–100. 26506661

[28] Gross K, Alba S, Glass TR, Schellenberg JA, Obrist B. Timing of antenatal care for adolescent and adult pregnant women in south-eastern Tanzania. BMC Pregnancy Childbirth. 2012;12:16 doi: 10.1186/1471-2393-12-16 22436344 10.1186/1471-2393-12-16 PMC3384460

